# Factors Predicting Diuretic Resistance in Patients With Acute Decompensated Heart Failure

**DOI:** 10.14740/jocmr6391

**Published:** 2026-04-15

**Authors:** Rarsari Soerarso, Dian Yaniarti Hasanah, Emir Yonas, Ahmad Pandu Pratama, Sunu Budhi Raharjo, Bambang Budi Siswanto, Maarten J.M. Cramer, Pim van der Harst, Marish I.F.J. Oerlemans

**Affiliations:** a Department of Cardiology and Vascular Medicine, Faculty of Medicine Universitas Indonesia, National Cardiovascular Center Harapan Kita, Jakarta 11420, Indonesia; b Department of Cardiology, University Medical Center Utrecht, University of Utrecht, Utrecht 3584, the Netherlands; c Division of Heart & Lungs, Department of Cardiology, University Medical Center Utrecht, University of Utrecht, Utrecht 3584, the Netherlands

**Keywords:** Heart failure, Systolic heart failure, Cardiorenal syndrome, Heart decompensation, Congestive heart failure

## Abstract

**Background:**

Acute decompensated heart failure (ADHF) is a leading cause of mortality and morbidity in the world. Diuretic resistance occurs in 20–30% of patients with ADHF and is an independent predictor of worsening clinical outcomes, immediate post-treatment death, and re-admission events. This study aims to: 1) identify factors that influence the occurrence of diuretic resistance in ADHF patients based on the underlying disease, comorbidities, vital signs, left ventricular ejection fraction, and laboratory parameters, and 2) investigate the clinical characteristics that serve as indicators of diuretic resistance incidence in patients with ADHF.

**Methods:**

A retrospective cohort study was conducted on 535 patients treated with ADHF during the period from January to December 2019. Diuretic resistance was defined as a diuresis response of less than 1,400 mL in the first 24 h after administration of 40 mg of intravenous (IV) furosemide (or equivalent). Subjects were observed for 24 h post 40 mg IV furosemide for occurrence of diuretic resistance. Bivariate and multivariate analyses were performed to synthesize clinical scoring system to predict occurrence of diuretic resistance.

**Results:**

Diuretic resistance occurs in 68% of patients. Independent predictors obtained from multivariate logistic regression analysis were: history of diabetes mellitus (DM, P = 0.013), history of using IV loop diuretics > 6 days (P = 0.002), oral loop diuretic dose > 80 mg/day (P = 0.006), left ventricular ejection fraction (LVEF) ≤ 49% (P = 0.002), blood urea nitrogen (BUN) ≥ 21 mg/dL (P < 0.001), and serum chloride < 98 mmol/L (P < 0.001). In addition, a scoring system has been made from the final model.

**Conclusion:**

DM, history of IV loop diuretic, daily loop diuretic dosage, LVEF < 49%, BUN > 21 mg/dL, and serum chloride < 98 mmol/L were found to be statistically significant in association with occurrence of diuretic resistance using multivariate analysis and can be synthesized into a clinical scoring system to help predict diuretic resistance.

## Introduction

Heart failure (HF) remains a significant public health issue with high prevalence and incidence in both developing and developed countries. HF also contributes to a significant portion of admitted patients in these countries. Even with the advancement in the treatment of acute decompensated heart failure (ADHF), the rehospitalization and mortality rates remain high. It is estimated that 29–50% of patients with HF will experience rehospitalization in a year, with an intrahospital mortality rate of 3–4% [1, 2].

The population of ADHF patients in Indonesia possesses a rather different clinical presentation and characteristics compared to developed countries. Patients in Indonesia commonly present with a younger age, more severe clinical presentation, lower ejection fraction, and the common finding of diabetes mellitus (DM) as a comorbidity [1]. The primary manifestation of acute or chronic HF is still retention and accumulation of fluids that lead to congestion at the organ level. Loop diuretics have been proven to be effective in alleviating congestion in patients with ADHF, as has been recommended by various treatment guidelines [3–5].

The long-term use of loop diuretics might lead to diuretic resistance. Diuretic resistance is a phenomenon in which impaired sensitivity to diuretics occurs, leading to reduced diuresis and natriuresis response, which impedes euvolemia status in patients with chronic HF [3]. Studies have shown diuretic resistance to be 20–30% in patients with HF; diuretic resistance has also been shown to be predictive of rehospitalization and death [6–8]. However, there have not been any clinical indicators of diuretic resistance in HF patients. This study aims to investigate the clinical characteristics that serve as indicators of diuretic resistance incidence in patients with ADHF.

## Materials and Methods

**Figure 1:**
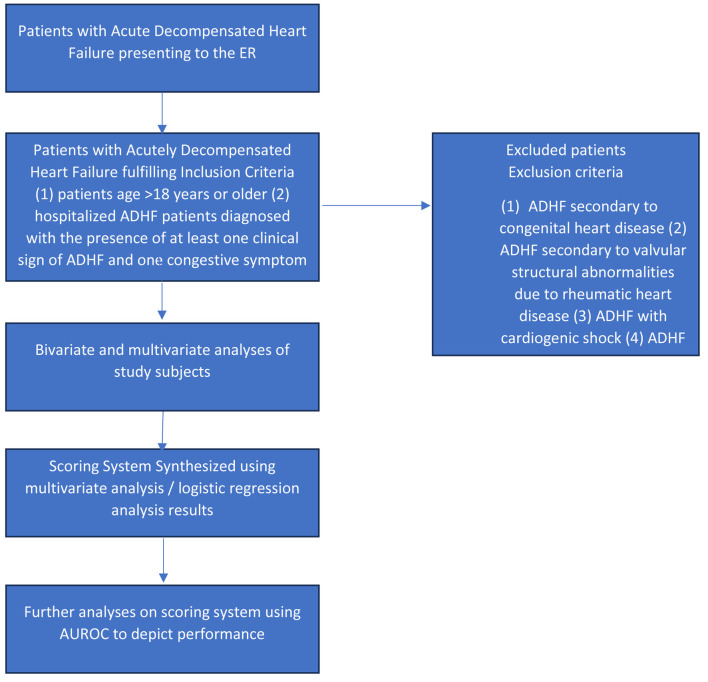
Study flowchart for patients selection and analysis.

This is a retrospective cohort study that included all patients diagnosed with ADHF who were hospitalized in National Cardiovascular Center Harapan Kita (NCCHK), Jakarta, Indonesia, between January 1, 2019, and December 31, 2019 (Fig. 1). Data were gathered using medical records. The inclusion criteria were: 1) patients aged > 18 years or older; 2) hospitalized ADHF patients diagnosed with the presence of at least one clinical sign of ADHF (dyspnea, orthopnea, or edema), and one congestive symptom of ADHF (basilar rales, peripheral edema, ascites, increased jugular venous pressure), or any signs of pulmonary congestion from a chest X-ray. The exclusion criteria were: 1) ADHF secondary to congenital heart disease; 2) ADHF secondary to valvular structural abnormalities due to rheumatic heart disease; 3) ADHF with cardiogenic shock; 4) ADHF patients with chronic kidney disease (CKD) with estimated glomerular filtration rate (eGFR) < 15 mL/min/1.73 m^2^.

Diuretic resistance was defined as a diuresis response of less than 1,400 mL after administration of 40 mg of intravenous (IV) furosemide equivalents in the first 24 h [7]. Diabetes is defined as a positive oral glucose tolerance test, HbA1c > 6.5%, or fasting plasma glucose > 126 mg/dL. CKD was defined as reductions in eGFR as per KDIGO recommendation, with GFR based on serum creatinine calculated using the CKD-EPI formula. Anamnesis, physical examinations, and laboratory panels were done in the emergency department at the patient’s initial presentation, while other data were retrospectively collected from medical records. Several numerical variables were transformed into categorical variables based on the current literature regarding diuretic resistance and ADHF. The sixth day of loop diuretic usage as an onset of diuretic resistance was chosen as threshold, based on several studies that observed hypertrophy and hyperplasia of renal distal convoluted tubule (DCT) and the occurrence of what is known as braking phenomenon that can be observed from the sixth day of loop diuretic administration [9–11]. Daily furosemide dose threshold of 80 mg is based on a study that defines diuretic resistance as persistent congestion necessitating hospitalization in patients already receiving daily doses of 80 mg of furosemide [12]. We elected to choose 49% as a threshold for ejection fraction in our study based on the guideline published by the European Society of Cardiology, which differentiates heart failure with reduced ejection fraction (HFREF) and heart failure with moderately reduced ejection fraction at 49% of ejection fraction [5]. Laboratory values were dichotomized based on established reference ranges.

This study was written as per the guidelines of Transparent Reporting of a multivariable prediction model for Individual Prognosis Or Diagnosis (TRIPOD) guideline [13].

### Data analysis

Categorical data were presented in frequency and proportion. Numerical data were evaluated with a normality test. Data were analyzed with IBM SPSS Statistics for Windows (Version 25.0; IBM Corp., Armonk, NY). Numerical data with normal distribution were presented in mean ± SD, while abnormally distributed numerical data were presented with median (min–max). Bivariate analyses were done using the Chi-square test and Fisher’s exact test between predictor variables of diuretic resistance. Multivariate analyses were done in bivariate variables with P values < 0.25. We performed logistic regression with the backward selection method. Results of the multivariate analysis were further tested with the Hosmer-Lemeshow test for calibration and receiver operating characteristics (ROC) for discrimination test, and optimal sensitivity and specificity were determined using ROC results (Fig. 1). The final multivariate model was transformed into a scoring system using the B coefficient and standard error values. Variables that were included in the scoring system were weighed using the B coefficient and standard error values and were reapplied to the overall study subjects. The final scoring system model was then analyzed against the outcome of the study using logistic regression to determine the incidence probability of the study outcome using the proposed scoring system. The proposed scoring system was then analyzed using the area under the curve (AUC) method to determine sensitivity, specificity, and cutoff value using optimum specificity and sensitivity values.

### Ethical clearance

Ethical clearance for this study was issued by the Institutional Ethical Review Board of NCCHK (Decision Letter Number LB.02.01/VII.450/KEP.055/2020).

## Results

A total of 535 patients were included in this study between January and December 2019 at NCCHK. Data from 579 samples were initially gathered from the hospital medical record system; 44 samples were excluded due to incomplete data. Baseline (bivariate) characteristics analyses were performed on all subjects. Multivariate analyses were done in bivariate variables with P-values < 0.25. We performed logistic regression, which was then used to construct a predictive model.

The study subjects consisted of ADHF patients; DM was found in 79.4% of the study subjects. Non-dialysis-dependent CKD was found in 6.9% of subjects. Diuretic resistance was found in 68.2% of study subjects. Due to the abnormal distribution of data, several variables were expressed in medians. There were 79.1% male subjects in this study, and no statistically significant difference in diuretic resistance was observed across genders. We observed higher medians of age in patients with diuretic resistance, and more patients with DM, CKD, use of loop diuretics > 6 days, and daily dose of loop diuretics > 80 mg (11.8% vs. 0.6%, P < 0.001) were found to have diuretic resistance. Lower systolic and diastolic blood pressure was also observed in patients with diuretic resistance compared to those without. On echocardiographic parameters, we observed higher medians of left ventricular ejection fraction (LVEF) and tricuspud annular point of systolic excursion (TAPSE) in the group without diuretic resistance. Most of the laboratory parameters in this study were observed to have a significant difference between groups with diuretic resistance and without diuretic resistance, such as blood urea nitrogen (BUN), serum creatinine, eGFR, serum sodium, and serum chloride (Table 1).

**Table 1:** Baseline Characteristics of Study Subjects

Variables	All (N = 535)	Patients with diuretic resistance (N = 365 (68.2%))	Patients without diuretic resistance (N = 170 (31.8%))	P-value
Age (years), median (min–max)	58 (18–91)	58 (18–81)	61 (20–91)	0.011
Gender				
Male, n (%)	425 (79.4%)	290 (79.5%)	135 (79.4%)	0.991
Female, n (%)	110 (20.6%)	75 (20.5%)	35 (20.6%)	
DM, n (%)	252 (47.1%)	191 (52.3%)	61 (35.9%)	< 0.001
CAD, n (%)	428 (80%)	289 (79.2%)	139 (81.8%)	0.486
CKD, n (%)	37 (6.9%)	31 (8.5%)	6 (3.5%)	0.035
Usage of IV loop diuretics > 6 days	216 ( 40.4%)	175 (47.9%)	41 (24.1%)	< 0.001
Oral loop diuretic dose > 80 mg/day, n (%)	44 (8.2%)	43 (11.8%)	1 (0.6%)	< 0.001
Clinical parameters				
SBP (mm Hg), median (min–max)	125 ± 24	122 ± 23	130 ± 25	< 0.001
DBP (mm Hg), median (min–max)	76 ± 15	75 ± 14	79 ± 16	0.006
LVEF (%), median (min–max)	25 (13–79)	23 (13–79)	29 (14–77)	< 0.001
TAPSE (mm), median (min–max)	16 (5–30)	15 (5–30)	17 (5–28)	0.019
Laboratory values				
Serum creatinine (mg/dL), mean (SD)	1.46 ± 0.63	1.54 ± 0.65	1.28 ± 0.57	< 0.001
GFR (mL/min/1.73 m^2^), median (min–max)	53 (16–146)	50 (16–132)	67 (17–146)	< 0.001
BUN (mg/dL), mean (SD)	25 ± 14	27 ± 14	20 ± 12	< 0.001
Serum sodium (mmol/L), median (min–max)	137 (111–150)	136 (111–144)	138 (118–150)	< 0.001
Serum potassium (mmol/L), mean (SD)	4.0 ± 0.69	4.1 ± 0.7	4.0 ± 0.6	0.192
Serum chloride (mmol/L), median (min–max)	100 (78–111)	98 (78–110)	102 (82–111)	< 0.001

BUN: blood urea nitrogen; DM: diabetes mellitus; CAD: coronary artery disease; CKD: chronic kidney disease; DBP: diastolic blood pressure; GFR: glomerular filtration rate; IV: intravenous; LVEF: left ventricular ejection fraction; max: maximum; min: minimum; SD: standard deviation; TAPSE: tricuspud annular point of systolic excursion.

Continuous variables were dichotomized before bivariate analysis based on median values and established clinical reference ranges. We then performed bivariate analysis between variables and incidence of diuretic resistance in patients, using Chi-square methods, and we calculated the odds ratio (OR), 95% confidence interval (CI), and P-value. P value of 0.25 was set as the threshold for inclusion in multivariate analysis (performed on all subjects, n = 535). Based on our results, variables that satisfy this threshold were age > 60 years (P = 0.011), DM (P < 0.001), CKD (P = 0.043), history of IV loop diuretic use > 6 days (P < 0.001), daily loop diuretic dose > 80 mg (P < 0.001), LVEF < 49% (P < 0.001), serum creatinine > 1.5 mg/dL (P < 0.001), eGFR < 59 mL/min/1.73 m^2^ (P < 0.001), BUN > 21 mg/dL (P < 0.001), serum sodium < 135 mmol/L (P = 0.002), and serum chloride < 98 mmol/L (P < 0.001) (Table 2). There were 198 patients (37%) who received a sub-maintenance dose of IV loop diuretics within 6 h of initial presentation, and 75.8% of these patients were diuretic-resistant (OR 1.773, 95% CI 1.196–2.629; P = 0.004).

**Table 2:** Bivariate Analysis Result

Variable	Diuretic resistance				P	OR	95% CI	
	Yes		No					
	N	%	N	%			Minimum	Maximum
Age								
> 58 years	194	72.4	74	27.6	0.041	0.679	0.471	0.980
≤ 58 years	171	64	96	36				
Gender								
Male	290	68.3	135	31.7	1.000	1.002	0.639	1.572
Female	75	67.9	35	32.1				
DM								
Yes	191	75.8	61	24.2	< 0.001	1.961	1.348	2.853
No	174	61.5	109	38.5				
CAD								
Yes	289	67.5	139	32.5	0.562	0.848	0.533	1.349
No	76	71	31	29				
CKD								
Yes	31	83.8	6	16.2	0.043	2.537	1.038	6.202
No	334	67	164	33				
History of IV loop diuretic use > 6 days								
Yes	175	81	41	19	< 0.001	2.898	1.929	4.354
No	190	59.6	129	40.4				
Oral loop diuretic dose > 80 mg/day								
Yes	43	97.7	1	2.3	< 0.001	22.568	3.081	165.322
No	169	34.4	322	65.6				
SBP								
< 90 mm Hg	18	75	6	25	0.654	1.418	0.553	3.639
≥ 90 mm Hg	347	68	164	32				
DBP								
< 60 mm Hg	42	65.6	22	34.4	0.668	0.875	0.504	2.998
≥ 60 mm Hg	323	68.6	148	31.4				
LVEF								
≤ 49%	348	70.2	148	29.8	0.001	3.016	1.581	5.756
≥ 50%	18	43.9	23	56.1				
TAPSE								
< 17 mm	209	72.1	81	27.9	0.041	1.472	1.021	2.122
≥ 17 mm	156	63.7	89	36.3				
Serum creatinine								
≥ 1.5 mg/dL	210	62.7	125	37.3	< 0.001	2.050	1.376	3.055
< 1.5 mg/dL	155	77.5	45	22.5				
GFR								
≤ 59 mL/min/1.73 m^2^	233	75	78	25	< 0.001	2.082	1.439	3.013
≥ 60 mL/min/1.73 m^2^	132	59	92	41				
BUN								
≥ 21 mg/dL	227	80	57	20	< 0.001	3.261	2.225	4.779
< 21 mg/dL	138	55	113	45				
Serum sodium								
< 135 mmol/L	122	78.2	34	21.8	0.002	2.008	1.301	3.100
≥ 135 mmol/L	243	64	136	36				
Serum potassium								
< 3.5 mmol/L	68	66	35	34	0.638	0.883	0.560	1.393
≥ 3.5 mmol/L	297	68.8	135	31.2				
Serum chloride								
< 98 mmol/L	158	83.6	31	16.4	< 0.001	3.422	2.202	5.319
≥ 98 mmol/L	207	59.8	139	40.2				

BUN: blood urea nitrogen; CAD: coronary artery disease; CI: confidence interval; CKD: chronic kidney disease; DBP: diastolic blood pressure; DM: diabetes mellitus; GFR: glomerular filtration rate; LVEF: left ventricular ejection fraction; TAPSE: tricuspud annular point of systolic excursion.

**Figure 2:**
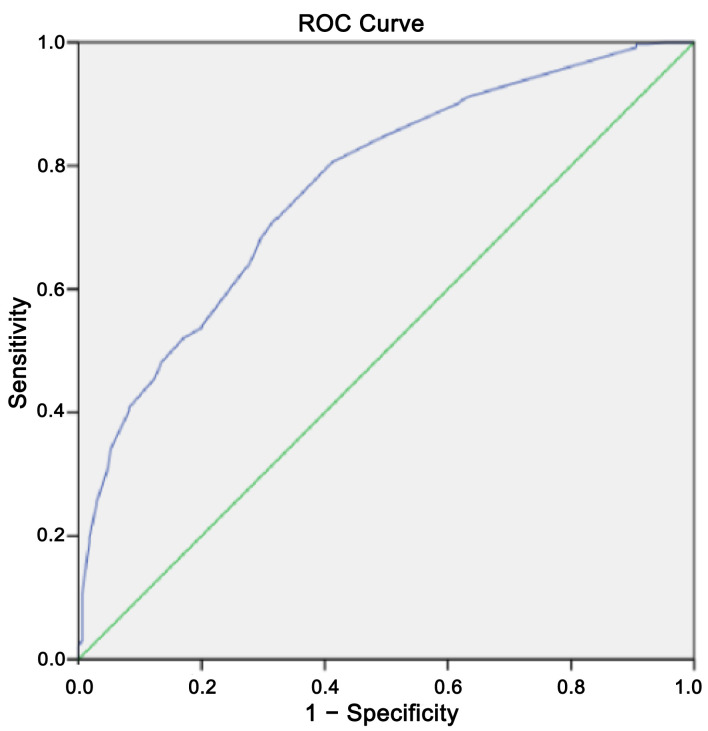
AUC result for the proposed diuretic resistance scoring system. AUC: area under the curve.

The final model of our multivariate analysis included DM, use of loop IV loop diuretics > 6 days, daily loop diuretic dose > 80 mg, LVEF < 49%, BUN > 21 mg/dL, and serum chloride < 98 mmol/L (Table 3). We performed the Hosmer-Lemeshow test on our scoring/prediction model, which generated a result of 0.823, a Chi-square value of 4.368 signifying a well-calibrated model that can be used to infer a causal relationship between independent and dependent variables, which we tested further using AUC model analysis, generating an AUC result of 0.767 (95% CI 0.725–0.809) (Fig. 2). In creating the scoring system, we performed weighing of variables in our final multivariate model using the B coefficient/standard error ratio (Table 4). These results were subsequently used to create a score weight for the presence of each corresponding variable in the final proposed scoring system (Table 5).

**Table 3:** Final Model of Multivariate Analysis

Variable	B	P value	Exp(B)	95% CI	
				Minimum	Maximum
Diabetes mellitus	0.528	0.013	1.696	1.120	2.570
History of IV loop diuretic use > 6 days	0.713	0.002	2.039	1.306	3.184
Daily loop diuretic dosage > 80 mg	2.839	0.006	17.107	2.265	129.177
LVEF ≤ 49%	1.159	0.002	3.186	1.530	6.633
BUN ≥ 21 mg/dL	0.907	< 0.001	2.477	1.641	3.740
Serum chloride < 98 mmol/L	0.993	< 0.001	2.699	1.672	4.357

B: coefficient B; BUN: blood urea nitrogen; CI: confidence interval; LVEF: left ventricular ejection fraction; SE: standard error.

**Table 4:** Scoring System Weighing

Variable	B	SE	B/SE	(B/SE)/2.491
Diabetes mellitus	0.528	0.212	2.491	1.0
History of IV loop diuretic use > 6 days	0.713	0.227	3.141	1.3
Daily loop diuretic dosage > 80 mg	2.839	1.032	2.751	1.1
LVEF ≤ 49%	1.159	0.374	3.099	1.2
BUN ≥ 21 mg/dL	0.907	0.210	4.319	1.7
Serum chloride < 98 mmol/L	0.993	0.244	4.070	1.6

B: coefficient B; BUN: blood urea nitrogen; LVEF: left ventricular ejection fraction; SE: standard error.

**Table 5:** Final Proposed Score Model

Variable	Score
Diabetes mellitus	1
History of IV loop diuretic use > 6 days	1
Daily loop diuretic dosage > 80 mg	1
LVEF ≤ 49%	1
BUN ≥ 21 mg/dL	2
Serum chloride < 98 mmol/L	2
Total score	8

BUN: blood urea nitrogen; LVEF: left ventricular ejection fraction.

Our final predictive model consists of: 1) DM, 2) history of IV loop diuretic use > 6 days, 3) daily loop diuretic dosage > 80 mg, 4) LVEF ≤ 49%, and 5) BUN ≥ 21 mg/dL.

The probability of diuretic resistance based on our proposed scoring system was calculated with linear regression with a specificity and probability threshold of 95%, based on the presence of predictive variables on the patient being assessed. The results showed a low risk of diuretic resistance on a score of 1–3 (39–65% probability), moderate risk of diuretic resistance on score 4–6 (76–91% probability), and high risk of diuretic resistance on score 7–8 (95–96.4% probability). The sensitivity and specificity for our current predictive models were 59.7% and 72.4%, respectively (Table 6).

**Table 6:** Scoring System Interpretation

Score	Criteria	Probability of diuretic resistance
1–3	Low risk	39–65%
4–6	Moderate risk	76–91%
7–8	High risk	95–96.4%

## Discussion

The main findings of this study are: 1) DM, history of IV loop diuretic, daily loop diuretic dosage, LVEF < 49%, BUN > 21 mg/dL, and serum chloride < 98 mmol/L were found to be statistically significant in association with occurrence of diuretic resistance using multivariate analysis. 2) Based on these findings, a scoring system with a fair performance (AUC 0.767 (0.725–0.809)) could be proposed to help quantitively predict the incidence of diuretic resistance in hospitalized ADHF patients.

In our study, diuretic resistance is defined as a urinary diuretic response of less than 1,400 mL per 40 mg of furosemide or equivalent. This definition was chosen based on a study on the ASCEND HF trial population by ter Maaten et al. The authors of this study concluded that an early assessment of diuretic response is as reliable as measurement after multiple days, thus providing clinical applicability in early identification of a diuretic-resistant patient [7, 14].

In this study, we observed a 68.2% prevalence of diuretic resistance out of 535 study subjects. This result is different from other studies regarding diuretic resistance in ADHF patients, which show a prevalence ranging from 20% to 35%. This finding might be caused by several factors such as the lower rates of loop diuretic administration in the first 6 h in this study and the different baseline characteristics of subjects in our study (198 patients (37%) received an initial sub-maintenance dose of IV loop diuretics within 6 h of initial presentation, 75.8% of these patients were diuretic-resistant (OR 1.773, 95% CI 1.196–2.629; P = 0.004)). This finding reflects a gap between guideline-directed medical therapy and real-world practices and challenges. We acknowledge that this inertia to fully implement guideline might mimic true diuretic resistance.

The higher rates of diuretic resistance in our study may also be caused by the advanced disease state of patients at presentation (median LVEF of 23% in diuretic resistance patients and median LVEF of 25% across all study subjects). Furthermore, higher diuretic resistance rates in our study subjects might also be caused by pseudo-resistance that is driven by initial suboptimal dosing. The European Society of Cardiology and the American Heart Association have recommended the administration of IV loop diuretics at 1–2.5 times the oral dose at the initial 6 h of presentation in patients with ADHF previously on oral loop diuretics [4, 15]. This recommendation stems from the decreased natriuretic and diuretic response to loop diuretics in a state of ADHF, which necessitates a higher loop diuretic dose. Inadequate IV dosing might even lead to decreased natriuretic and diuretic response, which will contribute to the incidence of diuretic resistance in ADHF patients [3].

Compared to previous studies on Western patients, we observed a lower median LVEF in our diuretic-resistant patients (23% (13–79%)). This finding is lower compared to patients’ LVEF in previous studies on ADHF diuretic resistance by Voors et al (37.9%), Valente et al (32.3%), and Trullas et al (50%). Compared to these studies, our subjects were also significantly younger with a median age of 58 years in the diuretic-resistant group, while the median age of patients was 72 years in the study by Voors et al, 70 years in the study by Valente et al, and 81 years in the study by Trullas et al [12, 16, 17]. The baseline characteristics of our study regarding age and LVEF were similar to a previous study by Siswanto et al which showed a trend of younger age (mean age of 60 years) and lower LVEF (mean LVEF of 33%) in Indonesian patients with ADHF, while patients in our country show lower trends of daily furosemide dose compared to Western countries [18]. While several other studies have studied the utilization of multiple agents to help curb diuretic resistance [14, 19], our study focused on synthesizing a new clinical scoring system using simple measurements to help better predict the occurrence of diuretic resistance in ADHF HFREF patients with type 2 DM. With early identification, diuretic-resistant patients might benefit from alternative treatment strategies to help overcome this resistance, ultimately leading to better outcomes for these patients.

More patients with diabetes developed diuretic resistance in our study. Diabetes has previously been known as a predictor of diuretic resistance [12, 17]. Diabetes causes increased RAAS activation, which necessitates the use of a higher loop diuretic dose to overcome this state. Insulin resistance in DM causes sodium retention in the kidneys through stimulation of Na^+^-K^+^-2CL^-^ and Na-K-ATPase cotransporters.

The use of loop diuretics causes specific changes in the microstructure of the kidneys. Hypertrophy and hyperplasia of the distal tubules of the kidneys have been found to occur on the sixth to eighth day of initial loop diuretic exposure; this phenomenon is known as a braking phenomenon [10, 11, 20]. In this study, based on this assumption, we found supporting findings that a history of diuretic dosage > 6 days is a predictor of diuretic resistance in ADHF patients. Theoretically, the braking phenomenon secondary to loop diuretic use is one of the contributing factors to diuretic resistance, which causes a positive correlation between loop diuretic dose and diuretic resistance onset. Although a history of diuretic usage > 6 days was identified as a strong predictor in our study, the retrospective nature of this study makes it rather challenging to establish definitive causality. Despite the fact that these findings are in accordance with the physiological “braking phenomenon” secondary to distal tubule hypertrophy, we acknowledge the possibility of reverse causality in which poorer diuretic response inadvertently necessitates longer decongestion efforts using loop diuretics. However, it should be noted that, regardless of this, a prolonged diuretic requirement is very useful to identify high-risk patients who might need more intense diuretic decongestion therapy.

A study by Trullas et al defined diuretic resistance as persistent congestion that necessitates hospital admission even with oral loop diuretics > 80 mg/day [12]. Based on our analysis, we found that the use of oral loop diuretics > 80 mg/day was strongly predictive of diuretic resistance (OR 15.107, 95% CI 2.265–129.177). The presence of DM as a comorbidity also poses a significant risk for diuretic resistance. This is due to the risk of nephropathy of DM itself; furthermore, a recent study has found a relationship between diabetic kidney disease and worsening in HF. In their study, Sharma et al described that microvascular derangements in both kidney and heart microvasculature might be the cause of increased risk of congestion in diabetic HF patients [21].

The study by Trullas et al also showed lower LVEF in patients with diuretic resistance [12]. The decreased cardiac output secondary to reduced LVEF activates RAAS, AVP, and the autonomic nervous system, which in turn causes retention of fluid and sodium, which ultimately dampens the kidney’s response to loop diuretics. In this study, we also found LVEF < 49% to be predictive of diuretic resistance (OR 3.186, 95% CI 1.530–6.633; P = 0.002).

BUN has been previously found to be a predictor of diuretic resistance [16, 17]. BUN has competitive properties against loop diuretics, especially furosemide, at the organic anion transporters (OAT) of the kidneys. The increase in BUN levels will cause reductions of binding between serum loop diuretics and OAT, which will contribute to the incidence of diuretic resistance. In this study, BUN > 21 mg/dL is found to be predictive of diuretic resistance (OR 2.477, 95% CI 1.641–3.740; P < 0.001). Hypochloremia is also found to be predictive of diuretic resistance in this study (OR 2.699, 95% CI 1.672–4.357; P < 0.001). Hypochloremia causes the release of renin and RAAS activation, which causes phosphorylation of with-no-lysine (WNK) kinase, which in turn will cause activation of Na^+^-K^+^-CL^−^ cotransporter that will reabsorb sodium and dampens effectivity of loop diuretics [22–24].

Although comorbidities commonly increase and worsen as age advances, we did not find age to be a predictor of diuretic resistance. Our age cutoff of 58 years was based on a previous study by Siswanto et al on the characteristics of Indonesian patients with ADHF. This caused a lower threshold of age compared to previous studies on ADHF [12, 16, 17].

We included TAPSE in our multivariate model to better reflect any alterations of right heart function in our study subjects, considering that right heart dysfunction was prevalent in patients with HFREF [25–27]. In this study, we observed 464 subjects (86.7%) with LVEF < 30% and 156 subjects (29%) with LVEF < 30% and criteria of advanced HF. However, TAPSE was not found to be statistically significant during our multivariate analysis (P = 0.787).

We did not find eGFR and serum creatinine to be statistically significant predictors of diuretic resistance in our multivariate analysis. This is similar to previous studies dealing with diuretic resistance and cardiorenal syndrome, which describe renal dysfunction in the spectrum of HF as a secondary condition to the worsening of heart function. Reduced eGFR and elevated serum creatinine as markers of kidney dysfunction in cardiorenal syndrome signify reduced renal perfusion, not a primary worsening of the kidneys. In patients with concurrent CKD and HF, the fall in eGFR was thought to result from a decline in cardiac output; an even further reduction in renal function might be seen in states of reduced renal perfusion, such as in ADHF before treatment [28, 29]. We did not find hyponatremia to be associated with diuretic resistance; this is also the finding of several previous studies [22–24]. In contrast with a previous study [16], we did not find hypokalemia to be predictive of diuretic resistance in our study.

There were more diuretic resistance incidences observed in patients with hypochloremia in this study (83.6% vs. 16.4%, P < 0.001). Our findings are similar to other studies in which hypochloremia is associated with diuretic resistance and increased mortality in ADHF patients [22, 30–33].

In ADHF patients, hypochloremia is commonly seen and associated with lower diuretic response and less decongestion. Decreased levels of serum chloride sensed by macula densa lead to reabsorption of electrolytes and fluid reabsorption through upregulation of NKCC and NCC receptors. With these receptors being the same receptors which loop diuretics act on, upregulation of these receptors leads to a blunted response to loop diuretics in HF patients [34]. Activation of this sodium retentive system occurs as a counter-regulatory response in ADHF patients treated with loop diuretics and causes significant changes in the pharmacokinetics of loop diuretics in ADHF [35]. This correlates with our multivariate model findings in which hypochloremia is associated with diuretic resistance (OR 2.699, 95% CI 1.672–4.357; P < 0.001).

With an AUC of 0.767, we expect our proposed scoring system to perform fairly in predicting the incidence of diuretic resistance. With this AUC value, this scoring system posed an acceptable discriminatory capability to predict the incidence of diuretic resistance at admission [36, 37]. However, we were unable to do a comparison since no other diuretic resistance predictive score has been proposed. A score of 5 in our scoring system was shown to be the point at which sensitivity and specificity balance (59.7% sensitivity and 72.4% specificity). We chose a probability threshold of 95% in determining high-risk criteria of diuretic resistance in our scoring system, which correlates to a score of 7–8 on the score (95% specificity). This scoring system is intended to help differentiate between diuretic responders and nonresponders at admission. In peripheral hospitals, this scoring system can help in early recognition of the need for referral in cases with anticipated diuretic resistance.

### Conclusion

DM, history of IV loop diuretic, daily loop diuretic dosage, LVEF < 49%, BUN > 21 mg/dL, and serum chloride < 98 mmol/L were found to be statistically significant in association with the occurrence of diuretic resistance using multivariate analysis. Based on these findings, a new scoring system with a fair performance could predict diuretic resistance among patients hospitalized with ADHF.

### Study limitations

This study has several limitations. First, our dataset does not include detailed information regarding previous routine medications of ADHF subjects, such as angiotensin receptor blockers, angiotensin-converting enzyme inhibitors, beta blockers, and mineralocorticoid receptor antagonists, which might influence diuretic responsiveness. Furthermore, our retrospective study utilized data from January to December 2019, which precedes the widespread recommendation and use of sodium-glucose cotransporter-2 (SGLT2) inhibitors for HF treatment. Consequently, SGLT2 inhibitor usage was negligible in our cohort and was not included as a variable. The presence of DM in this study was defined binarily; data regarding the profiling of long-term diabetic control and microvascular complications were not recorded during emergency admission. Specific laboratory parameters that dynamically reflect intravascular volume status, such as serial hematocrit or serum osmotic pressure, were not uniformly available in this retrospective dataset, limiting our ability to quantify baseline hydration status. As a retrospective observational study, our data reflect everyday clinical realities rather than the strictly controlled environment of trial conditions, and initial loop diuretic dosing was at the discretion of the treating physician; consequently we acknowledge that the clinical inertia regarding optimal initial loop diuretic dosing might mimic true physiological resistance; however, this inertia provides valuable insight to the challenges of an ideal implementation of HF guidelines. Finally, although our proposed scoring system demonstrates good calibration and fair discriminative capability (AUROC 0.767 and Hosmer-Lemeshow test), a further prospective validation in larger multicenter cohorts will be needed to further explore the clinical utility and generalizability of this scoring system.

## Data Availability

The datasets generated during and/or analyzed during the current study are available from the corresponding author on reasonable request.

## Abbreviations

ADHF: acute decompensated heart failure
AUC: area under the curve
BUN: blood urea nitrogen
CAD: coronary artery disease
CKD: chronic kidney disease
DBP: diastolic blood pressure
DM: diabetes mellitus
eGFR: estimated glomerular filtration rate
LVEF: left ventricular ejection fraction
ROC: receiver operating curve
TAPSE: tricuspud annular point of systolic excursion
